# Plasma calprotectin as infection biomarker in hip fracture patients: a prospective cohort study

**DOI:** 10.2340/17453674.2026.46719

**Published:** 2026-10-19

**Authors:** Helmi-Sisko PYRHÖNEN, Johan THAM, Anna STEFÁNSDÓTTIR, Alicia PERSSON, Linnea MALMGREN, Cecilia ROGMARK

**Affiliations:** 1 Department of Orthopedics, Skåne University Hospital, Malmö; 2 Department of Clinical Sciences, Faculty of Medicine, Lund University, Malmö; 3 Department of Infectious Diseases, Skåne University Hospital, Malmö; 4 Department of Translational Medicine, Lund University, Malmö; 5 Department of Orthopedics, Skåne University Hospital, Lund; 6 Department of Clinical Sciences, Lund, Orthopedics, Lund University; 7 Department of Geriatrics, Skåne University Hospital, Malmö, Sweden

## Abstract

**Background and purpose:**

We aimed to compare the diagnostic performance of plasma calprotectin in detecting infections following hip fracture surgery with 3 other biomarkers: procalcitonin (PCT), C-reactive protein (CRP), and white blood cell (WBC) count.

**Methods:**

This prospective cohort study included surgically treated hip fracture patients aged ≥ 65 years, admitted to our department from April 2023 to August 2024. Plasma calprotectin, PCT, CRP, and WBC count were measured on postoperative day 3. The outcome was any infection within 30 days (primary) and 7 days (secondary). Biomarker performance was evaluated using receiver operating characteristic analyses in a comparative framework.

**Results:**

166/550 individuals were analyzed (median age 85 years, 68% women). 19% (n = 31) and 12% (n = 20) had infection within 30 and respectively 7 days. For 30-day infections, calprotectin showed highest area under the curve (AUC) of 0.66 (95% confidence interval [CI] 0.54–0.77), followed by WBC count 0.60 (CCI 0.48–0.72), CRP 0.53 (CI 0.42–0.63), and PCT 0.51 (CI 0.40–0.63). Difference in AUC was largest between calprotectin and PCT (0.14, CI 0.00–0.29), followed by CRP (0.12, CI –0.01 to 0.26), while similar to WBC count (0.06, CI –0.05 to 0.16). For 7-day infections, AUCs were as following: calprotectin 0.70 (CI 0.58–0.82), WBC count 0.69 (CI 0.56–0.81), CRP 0.56 (CI 0.42–0.69), and PCT 0.48 (CI 0.33–0.63). Difference in AUC was largest between calprotectin and PCT (0.22, CI 0.05–0.39), followed by CRP (0.15, CI –0.02 to 0.31), while WBC count was similar (0.01, CI –0.09 to 0.12).

**Conclusion:**

Calprotectin demonstrated a trend towards superior discriminatory ability for infections compared with PCT and CRP, particularly for infections within 7 days. Calprotectin provided moderate diagnostic accuracy, likewise WBC count.

Hospitalizations for infectious diseases have increased substantially in recent decades, especially among older individuals [1,2]. Hospital-acquired infections may account for one-third of healthcare-related complications, with older surgical patients being overrepresented [3]. In orthopedic care, such infections are common and are associated with increased mortality and prolonged hospital stays [4-6].

Early diagnosis and treatment of bacterial infections are crucial to reduce morbidity, mortality, and healthcare costs. Yet diagnosing infections amongst old, often frail patients with hip fractures is challenging due to atypical presentations—such as delirium, postoperative fever unrelated to infection, or absent fever despite infection [7]. Conventional infection biomarkers like procalcitonin (PCT), C-reactive protein (CRP), and white blood cell (WBC) count elevate in response to surgical trauma itself, even in the absence of infection, complicating the timely and reliable detection of infection during the first postoperative days [8-11]. The unique course of hip fracture—initial fall trauma, potentially involving an underlying infection, followed by acute surgical intervention, and finally the high risk of infectious complications—highlights the need for a robust and rapidly responsive biomarker for infection in this vulnerable population.

Plasma calprotectin, a neutrophil activation marker [12], elevates rapidly in response to bacterial challenge in vitro [13]. It has shown promise in emergency and intensive care settings for identifying severe infections [14-17]. Calprotectin is validated for diagnosing chronic periprosthetic joint infection, primarily in synovial fluid [18] and less extensively in serum [19].

To our knowledge, plasma calprotectin’s diagnostic performance has not been studied regarding general infections after acute orthopedic trauma surgery. Based on the background described, we hypothesized its superiority to traditional biomarkers. Thus, we aimed to compare the diagnostic performance of plasma calprotectin, measured on the 3rd postoperative day, with that of PCT, CRP, and WBC count in detecting bacterial infections following hip fracture surgery.

## Methods

### Study design

This study was reported according to STROBE guidelines.

In this prospective cohort study, all patients aged 65 or older with acute, fall-related hip fracture admitted to the Department of Orthopedics at Skåne University Hospital in Malmö between April 15, 2023, and August 31, 2024, were included. Plasma calprotectin, PCT, CRP, and WBC count were measured on the 3rd postoperative day, along with other routine blood tests as per departmental protocol. Patients were followed for 30 days postoperatively to identify infections.

### Participants

All patients > 65 years, admitted to the Department of Orthopedics at Skåne University Hospital in Malmö after a fall-related hip fracture between April 2023 and August 2024, were included.

### Data preparation

Clinical data from the hospital stay, 30-day follow-up, and demographic variables was prospectively collected from medical records and entered into the REDCap electronic data capture tool (https://project-redcap.org/). All medical records were independently reviewed by at least 2 investigators. Quality checks included identification of missing data, diagnosis–treatment mismatches, and potential hazardous values. Microbiological culture results within 30 days were retrieved from the regional laboratory information system, which is shared across hospital and primary care settings.

### Outcome measures and covariates

Primary outcome was any bacterial infection (yes/no) diagnosed within 30 days post-surgery according to the pre-registered protocol. The secondary outcome, defined post-hoc, was infection within 7 days to better align with biomarker sampling timing.

Infections (pneumonia, UTIs, sepsis, superficial surgical site infections [SSIs], periprosthetic joint infections [PJIs], fracture-related infections [FRIs], and other soft tissue infections) were diagnosed by orthopedic surgeons or ward physicians in consultation with infectious diseases specialists or internal medicine specialists [20,21]. Microbiological confirmation was required for UTIs. Urine cultures were taken only if clinical signs raised suspicion of UTI, as our national guidelines on hip fracture treatment do not recommend routine UTI screening.

Data on the following covariates was collected: malignancy (yes/no; current or known history of cancer, excluding non-metastatic skin cancers), immunosuppression (yes/no; congenital immunodeficiency, HIV/AIDS, hematologic malignancies or immunosuppressive therapy), rheumatoid arthritis or inflammatory bowel disease (RA or IBD, respectively, yes/no), ASA score (dichotomized 1–2 vs 3–4), and surgery time (continuous) known at the index hospital admission. Covariates for the regression analyses described below were selected based on known risk factors from literature, clinical plausibility, and a directed acyclic graph (Supplementary Figure S1).

### Laboratory analyses

Blood samples were collected in lithium heparin plasma-separating tubes (calprotectin, CRP, and PCT), and EDTA tubes (WBC count). Calprotectin and CRP were analyzed by particle-enhanced immunoturbidimetry on Atellica (Siemens); PCT by Atellica IM BRAHMS PCT chemiluminescent immunoassay (Siemens); and WBC count by flow cytometry (Sysmex XN-10). Analyses were conducted at the accredited Department of Clinical Chemistry, Laboratory Medicine Skåne, Malmö, Sweden.

### Sample size

A 1-year study period was planned initially, thereafter to either incorporate or omit calprotectin in our clinical routine, depending on the study outcome. As we noted a noncompliance with the study protocol with missing sampling, we prolonged the study period by 6 months.

### Missing data

Participants with missing biomarker measurements were excluded from the main analyses, as the receiver operating characteristic (ROC) analysis requires complete data. 1 individual was missing ASA assessment and was excluded from the multivariable regression models.

### Statistics

Patient characteristics, treatments, and outcomes were summarized using mean and standard deviation (SD) for normally distributed, and median with interquartile range (IQ) for skewed variables.

The area under the ROC curve (AUC) with 95% confidence interval (CI) was calculated for each biomarker. Pairwise comparisons were performed using Delong’s method, with AUC differences and CIs derived from the variance-covariance matrix via Stata’s roccomp command. Primary comparisons were calprotectin vs PCT, CRP, and WBC count. All 6 possible pairwise comparisons among the 4 biomarkers were also performed and reported for completeness.

Sensitivity, specificity, positive predictive value (PPV), and negative predictive value (NPV) for each biomarker were calculated at the optimal Youden index.

Further, the association between biomarker levels and infection was studied in univariable (crude) and multivariable (adjusted for immunosuppression, IBD/RA, malignancy, ASA classification, and surgery time) binary logistic regression models. Separate regression models were conducted for each biomarker to avoid overadjustment. Biomarkers were analyzed as continuous variables and dichotomized at the optimal Youden index. No automated variable selection or internal validation was performed, as the study focused on diagnostic performance rather than prediction model development. Odds ratios with 95% CI are reported.

Statistical analyses were performed using Stata 19.5 (College Station, TX: StataCorp LLC; 2025).

### Ethics, registration, data sharing plan, funding, and disclosures

This study was conducted in accordance with the ethical principles of the Declaration of Helsinki and was approved by the Swedish Ethical Review Authority (2022-01997-01). Individuals were informed and could decline participation through an opt-out procedure. Oral and written study information was provided to patients and, when applicable, to legal representatives, who could decline participation. The pre-registered study protocol is available at ClinicalTrials.gov (NCT05712252).

Individual participant data that underlies the results reported in this study is not available due to restrictions in the ethical approval. Aggregated, group-level summaries may be provided upon reasonable request to the corresponding author. The analytical codes are available upon request.

This work was supported by research grants from Lions Research Foundation Skåne (Sweden) and a personal scholarship of the first author from Tegger Research Foundation (Sweden), used for laboratory costs and salaries. Disclosures: LM has received lecture fees from Amgen, CR from LINK and Swemac. Complete disclosure of interest forms according to ICMJE are available on the article page, doi: 10.2340/17453674.2026.46719

## Results

### Cohort characteristics

Of 657 individuals treated due to hip fracture during the inclusion period in our department, 107 were excluded for the following reasons according to the study protocol: admission not via the emergency department or > 24 h in another department (n = 44), no fall incident (n =1 8), fall > 7 days before admission (n = 18), residency outside Skåne region (n =1 1), patient opt-out (n = 1), and nonoperative treatment (n = 15, primarily isolated fractures of the greater trochanter). This resulted in 550 individuals meeting the initial eligibility criteria. Further, individuals with missing values for any of the 4 biomarkers (n = 384) were excluded. The final cohort comprised 166 patients (Figure 1).

**Figure 1:**
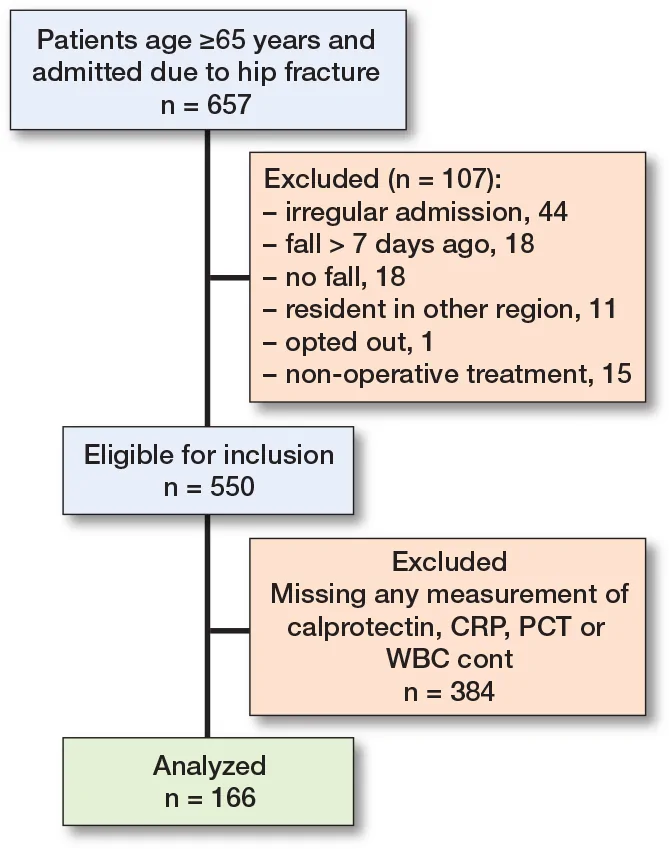
Flowchart illustrating the inclusion and exclusion of study participants. Patients with isolated greater trochanter fractures were managed nonoperatively

The baseline characteristics of the 166 included patients were comparable to the 550 eligible patients described in Table 1. The median age was 85 years, and 68% were women. 101 (61%) patients underwent internal fixation, and the remainder received arthroplasty (Table 1).

**Table 1:** Summary of baseline characteristics, fracture, and surgery details, in individuals with all biomarker measurements included in final analysis vs all eligible patients. Values are count (%) or as specified

Item	Included in final analyses (n = 166)	All eligible patients (n = 550)
Age, median (IQR)	85 (78–90)	85 (78–89)
Female sex	112 (68)	371 (68)
Smoker	22 (13)	67 (14)
ASA score 3–4	116 (70)	355 (66)
missing	1 (	11 (
Frail (CFS 5+)	78 (62)	238 (59)
missing	40 (	148 ( (
Chronic lung disease	26 (16)	79 (14)
Rheumatoid arthritis or IBD	10 (6.0)	30 (5.5)
Malignancy	12 (7.2)	53 (10)
Immunosuppression	14 (8.4)	36 (6.5)
Median surgery time, min (IQR)	79 (58–102)	78 (58–99)
Type of fracture		
Femoral neck	81 (49)	278 (51)
Intertrochanteric	69 (42)	223 (41)
Subtrochanteric	16 (10)	47 (8.5)
Pathological fracture	1 (0.6)	1 (0.2)
Periprosthetic fracture	3 (1.8)	5 (0.9)
Surgical treatment		
ORIF	101 (61)	334 (61)
Arthroplasty	65 (39)	216 (39)
Biomarker levels, median (IQR)		
Calprotectin (mg/L)	2.97 (2.07–3.98)	
WBC count (x 10⁹/L)	8.9 (7.3–11.0)	
PCT (µg/L)	0.23 (0.14–0.40)	
CRP (mg/L)	120 (72–191)	

ASA: American Society of Anesthesiologists Physical Status classification; CFS: Clinical Frailty Scale; CRP: C-reactive protein; IBD: inflammatory bowel disease; IQR: interquartile range; ORIF: open reduction and internal fixation; PCT: procalcitonin; WBC: white blood cell.

### 30-day infection

31 individuals (19%) had an infection within 30 days. Infections diagnosed were UTIs (n = 17), followed by pneumonia (n = 11), sepsis (n = 5), soft tissue infection (n = 2), and FRI (n = 1). 5 individuals had more than 1 type of infection. The median time from surgery to antibiotic start was 3 days; 8 patients received antibiotics before biomarker measurements (Supplementary Table S1A).

The AUC for calprotectin was 0.66 (CI 0.54–0.77), 0.60 (CI 0.48–0.72) for WBC count, 0.51 (CI 0.40–0.63) for PCT, and 0.53 (CI 0.42–0.65) for CRP. The difference between calprotectin’s vs other biomarkers’ AUC were as follows: (i) WBC count 0.06 (CI –0.05 to 0.16, P = 0.3), (ii) PCT 0.14 (CI 0.00–0.29, P = 0.057), and (iii) CRP 0.12 (CI –0.01 to 0.26, P = 0.7) (Figure 2, Table 2).

**Table 2:** Area under the curve (AUC) for each biomarker and pairwise comparison of the difference in AUC with 30-day infection and 7-day infection as outcome

Pairwise comparison	AUC1 (CI)	AUC2 (CI)	Difference in AUC (CI)	P value (Delong)
Infection 30 days				
Calprotectin – WBC	0.66 (0.54–0.77)	0.60 (0.48–0.72)	0.06 (–0.05 to 0.16)	0.3
Calprotectin – PCT	0.66 (0.54–0.77)	0.51 (0.40–0.63)	0.14 (0.00 to 0.29)	0.06
Calprotectin – CRP	0.66 (0.54–0.77)	0.53 (0.42–0.65)	0.12 (–0.01 to 0.26)	0.07
WBC – PCT	0.60 (0.48–0.72)	0.51 (0.40–0.63)	0.09 (–0.08 to 0.25)	0.3
WBC – CRP	0.60 (0.48–0.72)	0.53 (0.42–0.65)	0.07 (–0.09 to 0.22)	0.4
PCT – CRP	0.51 (0.40–0.63)	0.53 (0.42–0.65)	–0.02 (–0.15 to 0.11)	0.8
Infection 7 days				
Calprotectin – WBC	0.70 (0.58–0.82)	0.69 (0.56–0.81)	0.01 (–0.09 to 0.12)	0.8
Calprotectin – PCT	0.70 (0.58–0.82)	0.48 (0.33–0.63)	0.22 (0.05 to 0.39)	0.01
Calprotectin – CRP	0.70 (0.58–0.82)	0.56 (0.42–0.69)	0.15 (–0.02 to 0.31)	0.09
WBC – PCT	0.69 (0.56–0.81)	0.48 (0.33–0.63)	0.20 (0.02 to 0.39)	0.03
WBC – CRP	0.69 (0.56–0.81)	0.56 (0.42–0.69)	0.13 (–0.06 to 0.32)	0.2
PCT – CRP	0.48 (0.33–0.63)	0.56 (0.42–0.69)	–0.07 (–0.24 to 0.09)	0.4

CI: 95% confidence interval; CRP: C-reactive protein; PCT: procalcitonin; WBC: white blood cell.

**Figure 2:**
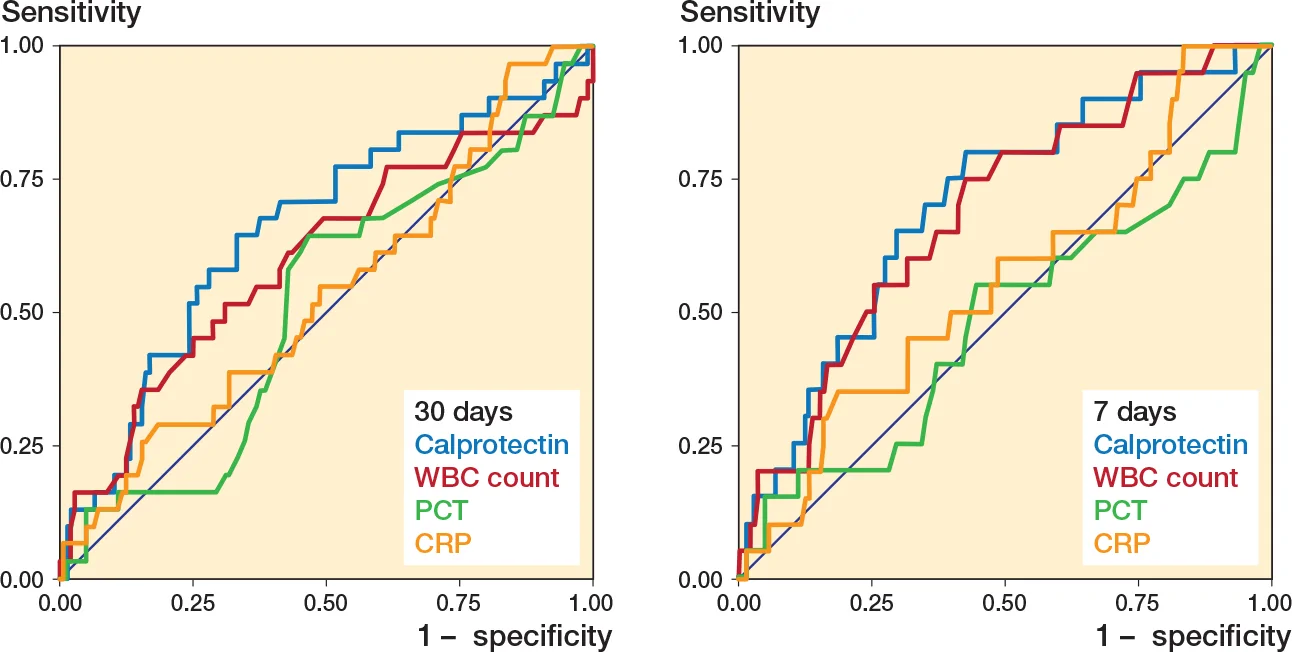
Receiver operating characteristic (ROC) curves of biomarkers for 30-day infection, and 7-day infection. CRP: C-reactive protein; PCT: procalcitonin; WBC: white blood cell.

At a cutoff ≥ 3.32 mg/L (highest Youden index), calprotectin had a sensitivity of 64% and specificity of 67%, while PPV was 29%, and NPV 87%. For comparison, WBC count at a cutoff ≥ 10.1 x 10^9^/L had a sensitivity of 52%, specificity 69%, PPV 28%, and NPV 86%. PCT and CRP presented high NPVs (87% and 96%), but low PPVs (24% and 21%), respectively (Supplementary Table S2A).

Univariable and multivariable logistic regression analyses (adjusted for immunosuppression, inflammatory disease, malignancy, and ASA score) are presented in Supplementary Table S3A. Calprotectin with cutoff ≥ 3.32 mg/L (OR 2.9, CI 1.2–6.7, P = 0.01) and WBC with cutoff ≥ 10.1 x 10^9^/L (OR 2.5, CI 1.1–5.9, P = 0.03) were independently associated with 30-day infection, while PCT (OR 2.0, CI 0.9–4.7, P = 0.1) and CRP (OR 4.2, CI 0.5–33.8, P = 0.2) presented with higher statistical uncertainty (Supplementary Table S3A).

### 7-day infection

20 individuals (12%) were diagnosed with infection within 7 days, of which 10 were UTIs, 9 pneumonias, 3 sepsis, and 1 soft tissue infection (Supplementary Table S1B).

Calprotectin had an AUC of 0.70 (CI 0.58–0.82), WBC count 0.69 (CI 0.56–0.81), PCT 0.48 (CI 0.33–0.63), and CRP 0.56 (CI 0.42–0.69). The difference between calprotectin’s vs other biomarkers’ AUC were as follows: (i) WBC count 0.01 (CI –0.09 to 0.12, P = 0.8), (ii) PCT 0.22 (CI 0.05–0.39, P = 0.1), and (iii) CRP 0.15 (CI –0.02 to 0.31, P = 0.09) (Figure 2, Table 2).

At a cutoff ≥ 3.08 mg/L (highest Youden index), calprotectin had a sensitivity of 80%, specificity 58%, PPV 20%, and NPV 94%. For WBC count, sensitivity was 75%, specificity 58%, PPV 20%, and NPV 94% at a cutoff ≥ 9.1 x 10^9^/L. PCT and CRP presented high NPVs (both 90%), but low PPVs (15% and 21%, respectively) (Supplementary Table S2B).

Univariable and multivariable logistic regression analyses (adjusted for immunosuppression, inflammatory disease, malignancy, and ASA score) are presented in Supplementary Table S3B. Calprotectin at a cutoff ≥ 3.08 mg/L (OR 4.4, CI 1.5–13.0, P = 0.008) and WBC count at a cutoff ≥ 9.1 x 10^9^/L (OR 4.1, CI 1.4–12.2, P =0 .01) were independently associated with 30-day infection, while PCT (OR 1.4, CI 0.5–3.7, P = 0.5) and CRP (OR 2.5, CI 0.9–7.1, P = 0.08) were not predictive (Supplementary Table S3B).

## Discussion

We studied the diagnostic accuracy of 4 inflammatory markers: plasma calprotectin, WBC count, PCT, and CRP as infection markers postoperatively in patients treated for acute hip fracture. Calprotectin demonstrated a trend toward superior discriminatory ability for infections compared with PCT and CRP, particularly for infections occurring within 7 days. Calprotectin, as well as WBC count, provided independent, moderate discriminatory ability for infection when measured on 3rd postoperative day. CRP and PCT showed no significant diagnostic value on bacterial infections. While all 4 biomarkers demonstrated excellent negative predictive values to rule out infection (86–96% for 30 days; 90–94% for 7 days), their ability to rule in infection remains low.

The results suggest that calprotectin and WBC count may be less influenced by surgical trauma than PCT and CRP on the 3rd postoperative day. This is in line with previous studies showing CRP and PCT peak at 2–3 days after surgery and infection [9], limiting their early diagnostic value in this setting. Hip fracture patients suffer the first trauma from the fall and fracture, and the second from surgery. As plasma calprotectin peaks earlier, the inflammatory effect might have subsided at the time of measurement on day 3, while CRP and PCT are still peaking, which could explain the results. We hypothesize that early de novo synthesis of calprotectin, along with elevated WBC count on day 3, may allow earlier detection of infection. Most infections were diagnosed on the 3rd postoperative day, which further underscores the need for better biomarkers than CRP or PCT.

Our AUC value for calprotectin on 30-day infection of 0.66 was lower compared with 2 studies on pneumonia/dyspnea patients and ICU patients, reporting 0.78 [17,22]. This may be explained by several factors, such as variations in patient populations, study designs, and clinical settings. Some participants may not have developed infection at the time of biomarker sampling, despite a median diagnosis time of 3 days. Restricting analysis to infections within 7 days slightly improved the AUC values but did not fully explain the lower diagnostic accuracy in patients with hip fracture. Also, early infections—possibly contributing to the initial fall—and prior antibiotic treatment may have suppressed postoperative biomarker levels. Our median calprotectin levels were higher than in previous studies, suggesting a notable influence of surgical and traumatic stress. However, we believe that calprotectin might be less obscured by surgical systemic inflammation than the conventional biomarkers.

Including UTIs may have contributed to the lower AUC values compared with studies focusing exclusively on pneumonia or sepsis. We included UTIs due to their clinical relevance in this cohort, being linked with sepsis and delirium [23,24]. While it can be argued that not all UTIs cause systemic inflammation detectable by biomarkers, it is often clinically challenging to distinguish early sepsis from localized infection. Also, pneumonia is largely a clinical diagnosis. Although most patients were tested for COVID-19, influenza, and respiratory syncytial virus (RSV), we cannot rule out that some cases may have involved viral infections.

### Limitations

To ensure a high participation rate, the study design was built on standard-of-care including an updated set of admission and 3rd-day blood tests. This design supported the ethics board’s approval of an opt-out consent procedure. On the other hand, relying on routine rather than research-dedicated sampling led to a 70% dropout rate due to missing biomarker data. Non-adherence to the new blood sampling routine was mainly caused by high staff turnover and workload. Baseline characteristics of analyzed patients were comparable to all eligible patients, supporting the assumption that missing cases occurred at random.

Further limitation of this study is the relatively small sample size and number of outcomes, increasing statistical uncertainty.

Infection diagnoses partly relied on unblinded biomarker results, which may have introduced positive bias for WBC count, CRP, and PCT, as clinicians are used to interpreting these postoperatively. Calprotectin results were available first on the day after sampling at earliest. Microbiological culture requests and results (e.g., urine, wound) were retrieved from the shared regional laboratory system used across hospital and primary care. However, infections diagnosed in primary care without cultures or laboratory requests may have been missed due to separate electronic health record systems. Further, the study design did not include repeated measures of the biomarkers.

### Strengths

This is, to our knowledge, the first study to evaluate plasma calprotectin in the context of acute orthopedic trauma and surgery. Calprotectin and WBC count consistently demonstrated a tendency toward higher diagnostic accuracy across several analyses, although these findings should be interpreted with caution given the limited precision of the estimates. The regression analyses suggested that higher calprotectin and WBC count were associated with an increased risk of infection, independent of the clinical covariates included in the models.

The inclusion of a broad range of postoperative bacterial infections, from pneumonia to surgical site infections, enhances the clinical relevance of our findings by reflecting the spectrum of infections encountered after hip fracture surgery.

### Clinical perspective

In addition to reducing morbidity and mortality, early and accurate infection diagnosis might shorten prolonged in-hospital stay associated with substantial healthcare costs and utilization of resources. Our study suggests plasma calprotectin may serve as a useful infection biomarker in older, surgically treated fracture patients, but did not offer a significant advantage over the cheaper WBC count. As no single biomarker performed excellently, further research should explore whether combining multiple biomarkers, with optimized cutoffs tailored to the specific timing after surgery, and clinical parameters can improve the overall diagnostic accuracy in postoperative settings. Further insight into calprotectin’s postoperative kinetics is essential to understand its clinical applicability in the future. Clinical assessment, together with monitoring using tools such as the National Early Warning Score (NEWS) and follow-up laboratory tests, remain essential for identifying individuals in the early stages of infections.

### Conclusion

Calprotectin demonstrated a trend toward superior discriminatory ability for infections compared with PCT and CRP, particularly for infections occurring within 7 days. Plasma calprotectin, as well as WBC count, provided an independent, moderate discriminatory ability for infection when measured on the 3rd postoperative day after hip fracture surgery. In contrast, single measurements of CRP and PCT did not show discriminatory ability for infections.

### Supplementary data

Figure S1 and Tables S1–S3 are available as supplementary data on the article page, doi: 10.2340/17453674.2026.46719

## Supplementary Material



## Notes

HSP: conceptualization; data curation; formal analysis; investigation; methodology; project administration; visualization; writing—original draft. AS, JT: conceptualization; supervision; writing—review & editing. AP: investigation; writing—review & editing. LM: conceptualization; methodology; supervision; visualization; writing—review & editing. CR: conceptualization; funding acquisition; investigation; methodology; project administration; resources; supervision; validation; writing—review & editing.

The authors would like to thank the FFF research team for their valuable contributions and the research administrator for their essential support throughout the study.

Handling co-editors: Per Henrik Randsborg and Robin Christensen

*Acta* thanks Alma B Pedersen for help with peer review of this manuscript.
