# Supplementary material for: Plasma calprotectin as infection biomarker in hip fracture patients: a prospective cohort study
Source: Acta Orthop. 2026 Oct 19;97:751–7. doi: 10.2340/17453674.2026.46719 (PMC13678456; doi:10.2340/17453674.2026.46719)
Supplement: Supplementary file 1 [file ActaO-97-46719-s1.pdf]

Supplementary data

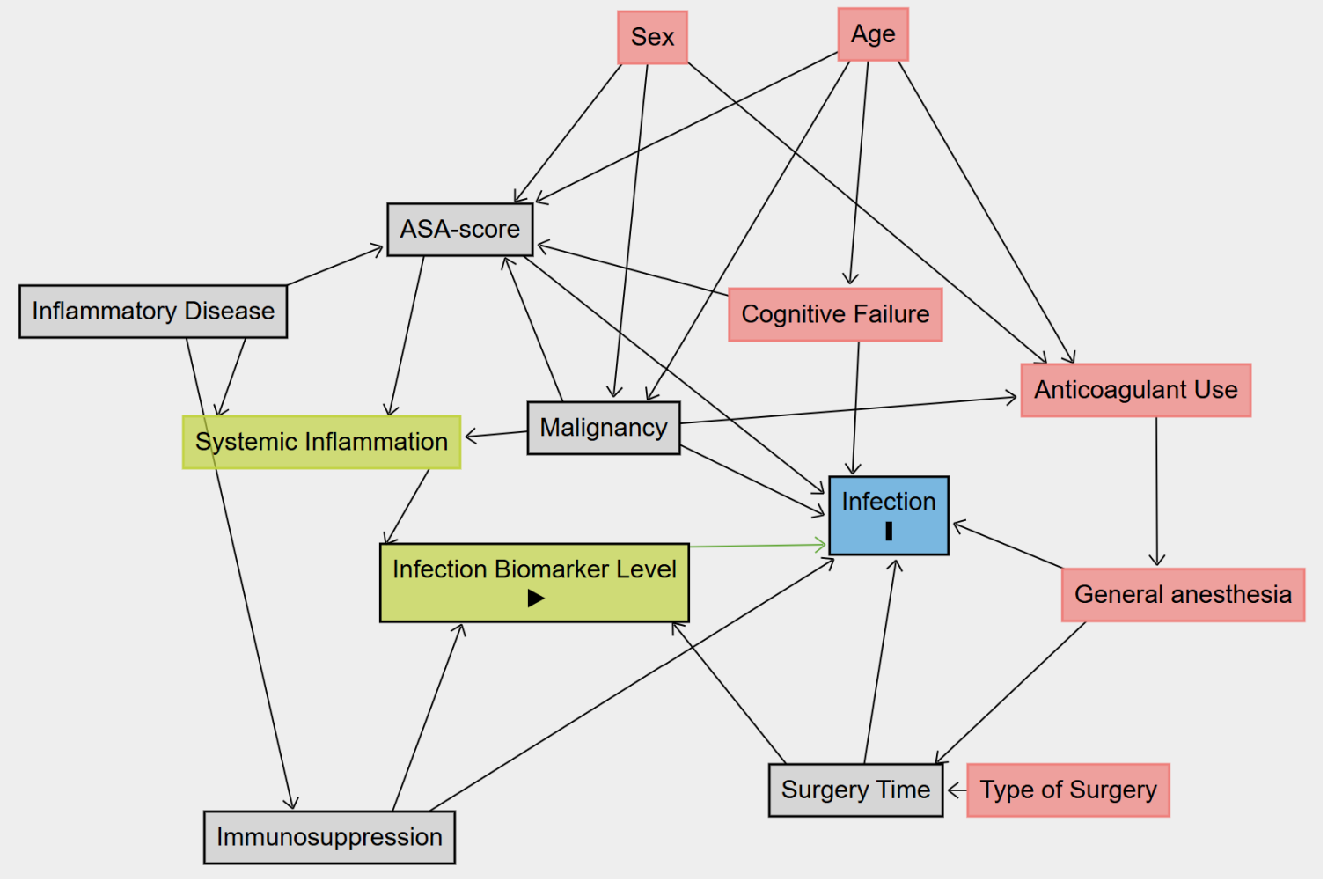

Supplementary Figure S1. A directed acyclic graph (DAG) illustrating the associations between potential confounders, biomarker level as the exposure, and infection as the outcome. The minimal sufficient adjustment set included inflammatory disease, ASA score, malignancy, immunosuppression, and surgery time.

Supplementary Table S1. Summary of infection details stratified by 30-day and 7-day infection status

30-day infection (n = 31)	
Days from surgery to antibiotic start, median (IQR)	3 (–1 to 10)
Type of infection [a], n	
UTI	17
Pneumonia	11
Sepsis	5
Other soft tissue infection	2
FRI	1
Superficial SSI	0
PJI	0
7-day infection (n = 20)	
Days from surgery to antibiotic start, median (IQR)	1 (–1 to 3)
Type of infection [b], n	
UTI	10
Pneumonia	9
Sepsis	3
Other soft tissue infection	1
FRI	0
Superficial SSI	0
PJI	0

[a] 1 had both FRI and sepsis, 1 both pneumonia and sepsis, 2 both UTI and sepsis, 1 both UTI and pneumonia.

[b] 1 had both pneumonia and sepsis, 1 had UTI and sepsis, 1 had UTI and pneumonia.

IQR: interquartile range, FRI: fracture-related infection, PJI: prosthetic joint infection, SSI: surgical site infection, UTI: urinary tract infection.

Supplementary Table S2. Sensitivity, specificity, positive predictive values (PPV), and negative predictive values (NPV) at the optimal Youden Index for each biomarker, with 30-day infection and 7-day infection as outcome

	Cut-off	Sensitivity	Specificity	PPV	NPV
Infection within 30 days					
Calprotectin	≥ 3.32 mg/L	0.64	0.67	0.29	0.87
WBC count	≥ 10.1 x 10^9/L	0.52	0.69	0.28	0.86
PCT	≥ 0.23 µg/L	0.65	0.53	0.24	0.87
CRP	≥ 51 mg/L	0.97	0.16	0.21	0.96
Infection within 7 days					
Calprotectin	≥ 3.08 mg/L	0.80	0.58	0.20	0.94
WBC count	≥ 9.1 x 10^9/L	0.75	0.58	0.20	0.94
PCT	≥ 0.25 µg/L	0.55	0.56	0.15	0.90
CRP	≥ 212 mg/L	0.35	0.82	0.21	0.90

CRP: C-reactive protein; PCT: procalcitonin; WBC: white blood cell.

Supplementary Table S3. Univariable (crude) and multivariable (adjusted) logistic regression analyses of each biomarker with 30-day infection and 7-day infection as binary outcome

	Crude OR (CI)	P value	Adjusted OR [a] (CI)	P value
	n = 166		n = 165	
30-day infection				
Biomarkers (continuous, per unit increase)				
Calprotectin (mg/L)	1.22 (1.02–1.46)	0.03	1.22 (1.02–1.48)	0.03
WBC count (x 10^9/L)	1.12 (1.01–1.24)	0.04	1.12 (1.00–1.24)	0.04
PCT (µg/L)	0.99 (0.96–1.03)	0.8	1.00 (0.96–1.03)	0.8
CRP (mg/L)	1.00 (1.00–1.01)	0.5	1.00 (1.00–1.01)	0.5
Biomarkers (dichotomized)				
Calprotectin ≥ 3.32 mg/L	2.8 (1.3–6.2)	0.01	2.9 (1.2–6.7)	0.01
WBC count ≥ 10.1 x 10^9/L	2.4 (1.1–5.2)	0.03	2.5 (1.1–5.9)	0.03
PCT ≥ 0.23 µg/L	2.1 (0.9–4.7)	0.08	2.0 (0.9–4.7)	0.1
CRP ≥ 51 mg/L	5.5 (0.7–42.8)	0.1	4.2 (0.5–33.8)	0.2
7-day infection	n = 166		n = 165	
Biomarkers (continuous, per unit increase)				
Calprotectin (mg/L)	1.26 (1.04–1.52)	0.02	1.27 (1.04–1.55)	0.02
WBC count (x 10^9/L)	1.18 (1.05–1.33)	0.006	1.18 (0.13–6.2)	0.007
PCT (µg/L)	1.00 (0.96–1.04)	>0.9	1.00 (0.97–1.04)	>0.9
CRP (mg/L)	1.00 (1.00–1.01)	0.4	1.00 (1.00–1.01)	0.4
Biomarkers (dichotomized)				
Calprotectin ≥ 3.08 mg/L	4.2 (1.4–12.1)	0.008	4.4 (1.5–13.0)	0.008
WBC ≥ 9.1 x 10^9/L	4.1 (1.4–11.8)	0.01	4.1 (1.4–12.2)	0.01
PCT ≥ 0.25 µg/L	1.5 (0.6–3.9)	0.4	1.4 (0.5–3.7)	0.5
CRP ≥ 212 mg/L	2.4 (0.9–6.5)	0.09	2.5 (0.9–7.1)	0.08

Each biomarker was analyzed in a separate model.
[a] Model was adjusted for immunosuppression, inflammatory bowel disease/rheumatoid arthritis, malignancy, American Society of Anesthesiologists classification, and surgery time.
Abbreviations: see Supplementary Table S2.
